# Applied sonoanatomy of the posterior triangle of the neck

**DOI:** 10.4103/0973-6042.76963

**Published:** 2010

**Authors:** Barys Ihnatsenka, André P Boezaart

**Affiliations:** 1 Department of Anesthesiology, Division of Acute Pain Medicine and Regional Anesthesia, University of Florida College of Medicine, Gainesville, Florida, USA; 2 Department of Orthopaedic Surgery and Rehabilitation, University of Florida College of Medicine, Gainesville, Florida, USA

**Keywords:** Brachial plexus block, cervical plexus block, neck, sonoanatomy, stellate ganglion block, supraclavicular area, thoracic outlet, ultrasound

## Abstract

The posterior triangle of the neck is an area of the body frequently visited by regional anesthesiologists, acute and chronic pain physicians, surgeons of all disciplines, and diagnosticians. It houses the entire brachial plexus from the roots to the divisions, the scalene muscles, the cervical sympathetic ganglions, the major blood vessels to and from the brain, the neuroforamina and various other structures of more or less importance to these physicians. Ultrasound (US) offers a handy visual tool for these structures to be viewed in real time and, therefore, its popularity and the need to understand it. We will discuss pertinent clinical anatomy of the neck and offer a basic visual explanation of the often-difficult two-dimensional (2-D) images seen with US.

## INTRODUCTION

The posterior triangle of the neck is an area of the body frequently visited by regional anesthesiologists, acute and chronic pain physicians, surgeons of all disciplines and diagnosticians. It houses the entire brachial plexus (BP), from the roots to the divisions, the scalene muscles, the cervical sympathetic ganglions, the major blood vessels to and from the brain, the neuroforamina and various other structures of more or less importance to these physicians. Ultrasound (US) offers a handy visual tool for these structures to be viewed in real time and, therefore, its popularity and the need to understand it. There are several components that are important for successful US image acquisition and understanding: (1) basic knowledge of “US appearance” of different tissues and anatomical structures, (2) knowledge of how adjustments to US machine settings and the use of different transducers may affect the US image to our best advantage, (3) knowledge of how transducer manipulation may help the ultrasonographer to get the best picture and how to understand US picture changes related to probe manipulation and (4) knowledge of clinical three-dimensional (3D) anatomy of the scanned area.

The previous article (“Ultrasound: Basic Understanding and Learning the Language”) is focused on the first three components. The purpose of this paper is to cover the last component. We will discuss pertinent clinical anatomy of the neck and offer a basic visual explanation of the often-difficult two-dimensional (2-D) images seen with US. The authors suggest that the accompanying article be studied before reading this paper. At the end of this article, we provide an example of a systemic approach to the use of US where we combine ideas from two papers.

### Clinical anatomy of the posterior triangle of the neck

#### Surface landmarks[1–6]

Knowledge of surface landmarks is useful for the initial probe placement on the skin or as a reference tool if the ultrasonographer “gets lost in the US image” and needs to regain his or her bearing.

- The posterior border of the posterior triangle of the neck is the trapezius muscle, its anterior border is the sternocleidomastoid muscle and its inferior border is the clavicle.
- The level of the cricoid cartilage corresponds to the level of the 6^th^ cervical vertebra (C6). This line is perpendicular to the spine and this must be kept in mind with flexion and extension of the neck.
- The external jugular vein commonly crosses the posterior border of the sternocleidomastoid muscle at the level where the BP exits between the anterior and the middle scalene muscles. This is commonly known as “Winnie’s point.”[5–7]
- The level of the upper margin of the thyroid cartilage corresponds to C4. The level of the chin with the head in neutral position also corresponds to C4.
- Dividing the distance between the C4 level and the tip of the mastoid process into thirds will indicate the positions of C2 and C3 on the line connecting the transverse process of C6 (carotid tubercle) and mastoid process if the neck is in the neutral position.
- The midpoint of the distance between the proximal and the distal attachments of the sternocleidomastoid muscle on its posterior border corresponds to the exit point of the superficial cervical plexus (SCP).
- The lateral border of the first rib is usually located on the line drawn through a point 3 cm lateral to the insertion point of the clavicular head of the sternocleidomastoid muscle. Cupola of the lung is logically located medial to the medial border of the rib.
- The midpoint of the clavicle usually corresponds to the position of the BP beneath it, although the position of the clavicle changes significantly with changes in the shoulder position.

### Cervical vertebrae, first rib and clavicle

Bones are not as well visualized on US as with fluoroscopy, but they are still visible (especially outer contour and, especially, if they are not too deep) and could be very helpful landmarks.

Even though it is not related to sonoanatomy it is important to know the 3D anatomy of the bones because they serve as protective barriers for some vital vascular and neural structures whenever needles are inserted in that area. Therefore, we dedicate some extra attention to the skeletal anatomy of the neck area. Aligning the target structure (nerve plexus for example) on the US screen in a way that other vulnerable structures (vertebral artery or neuraxium for example) are “shielded” by bone adds extra safety to the procedure in case the ultrasonographer loses the tip of the needle from the US view.

There are seven cervical vertebrae. The first (C1) does not have a typical spinous process, and the C2–C7 spinous processes are often split at the end.

The laminae of the cervical vertebrae is visible in the posterolateral sagital US plane and can be used for guiding a needle for placement on the bone to initiate a paramedial approach for the cervical intralaminar epidural.

The articular processes of the cervical vertebrae create articular “columns” with an uninterrupted bony wall that can also be seen in axial and sagital posterolateral views (personal observation). It is important to notice that from C3 to C6, the lateral border of the articular column is about 5–7 mm more lateral than the vertebral artery. That makes posterior approaches to the cervical or BP, such as Pipa’s or Boezaart’s approaches, safer in regard to inadvertent injury to the vertebral artery.[8,9] Changing needle trajectory from the true posterior–anterior, which gives “bony wall” protection of the articular column, to any other trajectory with needle direction from posterolateral to anteromedial makes the vertebral artery more vulnerable unless constant direct visualization of the needle and artery is used.

Facet joints are visible on US and can be used for facet joint injection. The “waist” of the articular process of each vertebra is also visible and is a surrogate marker for medial branch nerve block for diagnosis or treatment of facet joint arthropathy.

The transverse processes of the cervical vertebrae are different from other transverse processes.[1–4] A typical cervical transverse process is directed downward (caudad) and outward (anteriorly) and is shaped like a trough. Each of the transverse processes has an anterior tubercle and posterior tubercle, except the transverse process of C7, which has no or only a rudimentary anterior tubercle.

If the cervical spine is viewed from the side (lateral axial view), you should note that the transverse processes are not exactly underneath each other if the probe is moved down or up along the straight line because of the curvature of the neck (personal observation). When viewing the vertebrae from the side, it can be seen that the posterior tubercle is usually lower than the anterior tubercle of the upper cervical vertebrae (C2–C5). The lower vertebrae, especially C7, are the other way round. To visualize both tubercles, mild probe rotation may be needed.

The anterior tubercle of the C6 transverse process is prominent (carotid or Chassaignac’s tubercle).[1] It is an important landmark.

**Figure 1:**
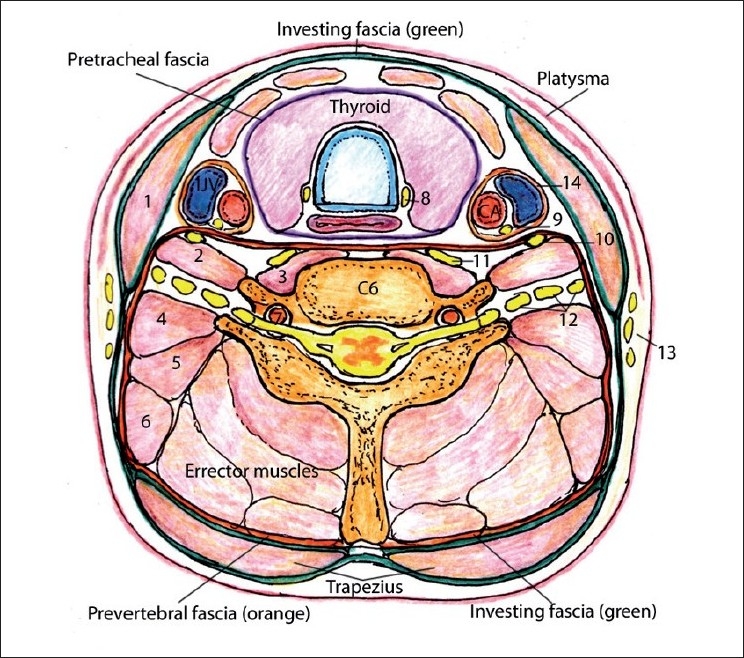
Axial cut through the neck at the C6 level. Fascias, muscles, bones, vessels and nerves: (1) sternocleidomastoid muscle, (2) anterior scalene muscle, (3) longus colli muscle, (4) middle scalene muscle, (5) posterior scalene muscle, (6) levator scapulae muscle, (7) vertebral artery, (8) recurrent laryngeal nerve, (9) vagus, (10) phrenic nerve, (11) sympathetic ganglion, (12) brachial plexus, (13) branches of superficial cervical plexus (supraclavicular nerves), (14) carotid sheath

**Figure 2:**
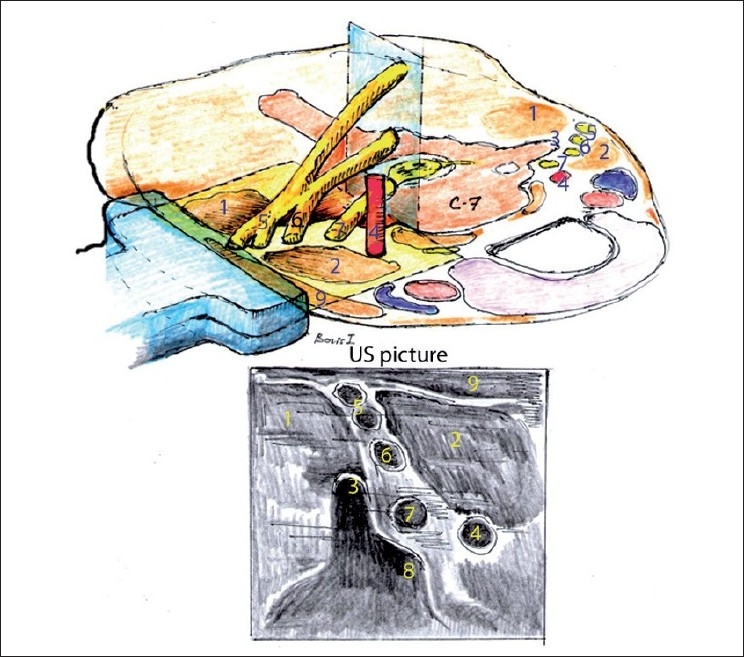
Schematic illustration of lateral axial ultrasound neck image generation at the C7 vertebral level. (1) Middle scalene muscle, (2) anterior scalene muscle, (3) posterior tubercle of C7, (4) vertebral artery, (5) derivates of the C5 roots, (6) C6 root, (7) C7 root, (8) rudimentary anterior tubercle of the C7 transverse process. Note the difference of the shape of the transverse processes of C6 [Figure 1] and C7 [Figure 2]

The C7 has the most distinguished shape. The C7 transverse process is usually positioned slightly more posterior and caudat than the C6 transverse process and has a big posterior tubercle and rudimentary or absent anterior tubercle when viewed in the axial view, which gives it a “thumb up” appearance on the US [Figures 1 and 2].

Determining both the C6 and the C7 transverse processes improves the specificity in determining the correct level of the cervical spine being viewed. To determine it at the higher level, one can slowly slide the probe cephalad while counting transverse processes from the known reference points of C7 and C6.

If using bony landmarks to determine the level of the cervical spinal nerve, it should be noted that the BP roots are situated between the anterior and the posterior tubercles of the corresponding transverse processes (C6 root at the C6 transverse process). The C8 root is, however, situated below the C7 transverse process and the T1 root originates from below the first rib (below the T1 transverse process).

All seven cervical vertebrae have transverse foraminae in their transverse processes.[1] The vertebral artery enters the C6 transverse foramen in 90% of the people. In 10% of the people, it enters the transverse foramen of C5 or C4.[10] The reason for the presence of a transverse foramen in the transverse process of the C7 vertebra is not fully understood.

The neuroforaminae are posterior and cephalad to the transverse process. It is important to study the position of the neuroforaminas because they could theoretically be the inadvertent entry point inside the spinal canal for the long block needles when the classic Winnie interscalene block (ISB) is performed.[11–13]

The classical Winnie’s recommendation for needle direction during ISB is “perpendicular to all planes with slight caudal angulation.” Caudad needle angulation theoretically was supposed to be safer than the horizontal direction. This was based on the fact that transverse processes also have some caudal angulation and the assumption that if the nerve root is missed (no paresthesia or motor response with needle stimulation) and the needle is being advanced medially, it would eventually hit the “floor” of the transverse process and would less likely enter the spinal canal via the neuroforamina. In reality, because the transverse foramen creates a “hole” in the “floor” of the transverse process, needle passage from a cephalad direction may not be interrupted by expected bone, and the needle will pass into the spinal canal via the neuroforamina below (personal observation on the skeleton).

Despite the above reasoning, some caudal angulation during needle advancement is probably safer than true horizontal needle direction (possible needle advancement straight into the neuroforamina at the level of entry). Theoretically based on analysis of the cervical spine anatomy, the most dangerous needle direction during classic Winnie’s ISB in regard to inadvertent entry into the spinal canal would be medial, posterior and cephalad. The distance from the skin to the spinal canal could be very small (about 2 cm) in some patients,[5] and even short needles may not protect from this catastrophic complication.

Posterior approaches to brachial and cervical plexuses,[8,9] or so called “lateral” approach,[14] are much safer in regard to inadvertent entry into the spinal canal compared with the classic Winnie approach. Visualization of the needle and the targeted structure with US makes posterior and lateral techniques even safer.

The anterior surface of the vertebral body and the intervertebral disks can also be visualized with US during an anterior sagital scan.

The first rib is angulated downward and anterior and it can be readily traced with US (personal observation). Slightly tilting and rotating the probe can maintain the vision of the first rib in the true axial view when tracing it from the head of the rib toward the sternum. Bony structures of the cervical spine could be used as a “back stop” for inadvertent injury to the vertebral artery and neuraxium by the block needle. The first rib could be used as a “back stop” for inadvertent injury to the pleura and lung during supraclavicular BP block.

The clavicle is an important bony structure that is fixed at the sternum and is mobile laterally when changing the position of the shoulder. This mobility could be used for obtaining more favorable scanning or needling window by moving the patient’s shoulder in the most optimal direction. This simple trick is frequently overlooked by practitioners (personal observation).

### Muscles and fascia

Visualizing and differentiating the muscle and fascial planes is important for intramuscular injection of botulinum toxin or is more commonly needed as important landmarks and reference points in our search for nerves during neural blockade. Commonly, small nerves are not seen with US and compartment blocks are performed. Therefore, knowledge of the sonoanatomy of muscle and fascial planes is very essential [Figure 1].

A hyperechoic rim surrounding the muscle represents fascia. Fascia is important because it influences the extent of LA spread.

(Additional comment to Figure 1) Note that the transverse process of C6 vertebrae has anterior and posterior tubercle. Figure 2 demonstrates an axial section of the neck at the C7 level, and you can see that the transverse process of C7 has no anterior tubercle and thus has a “thumb up” appearance on US.

The concept of BP sheath and plexus anesthesia is another example of the importance of fascia in regional anesthesia.[15–17] The “plexus sheath” remains a controversial concept and is looked at not as a specific tubular fascial structure

surrounding the neurovascular bundle but rather as a fascial compartment formed by the fascia of the surrounding muscles. The pattern of spread of LA around the nerve structures is readily observed during live US-guided nerve blocks. When observing the spread of LA during neural blockade using live US, you should remember that while you see only a 2D picture, in reality, the “pool” of LA has a 3D shape. Moving the US probe around may help to estimate the spread of LA more accurately.

The SCP (cutaneous innervation) is situated outside the prevertebral fascia (13 on Figure 1) of the neck and most of the nerves exit posterior of the midpoint of the sternocleidomastoid muscle.[1] It originates from the deep cervical plexus (DCP), which originates from the anterior rami of the C1–C4 spinal nerves.

The DCP is located below the prevertebral fascia in the paravertebral space at the level of C1–C4 and innervates the deep neck muscles via corresponding short branches and diaphragm via the phrenic nerve.[1] The BP originates from the anterior rami of C5–T1spinal nerves and is also located below the deep fascia in the paravertebral space, but more caudally.[1] Accidentally penetrating the prevertebral fascia during intended SCP block for cutaneous anesthesia or analgesia may unintentionally lead to a partial DCP block with resulting possible phrenic nerve paralysis or partial BP block and weakness of the arm (personal observation). The intentional blockade of the DCP in turn will also cause blockade of the SCP.[5]

Also note that the 11^th^ cranial nerve (the accessory nerve) is situated above the deep fascia in proximity to the SCP. This nerve could also be blocked during attempted SCP block, but is unlikely during a DCP block.[1–5]

Fascial planes are seen best during US-guided “hydro dissection.”

Adipose and soft connective tissue and lymph nodes are commonly seen between fascial planes and can be easily visualized by US to estimate their involvement in the pathologic processes (degree of lymphadenopathy for example). Occasionally, small deep lymph nodes may confuse the ultrasonographer because they may look like nerves (round hypoanechoic structures).

### Deep muscles of the neck

**Figure 3:**
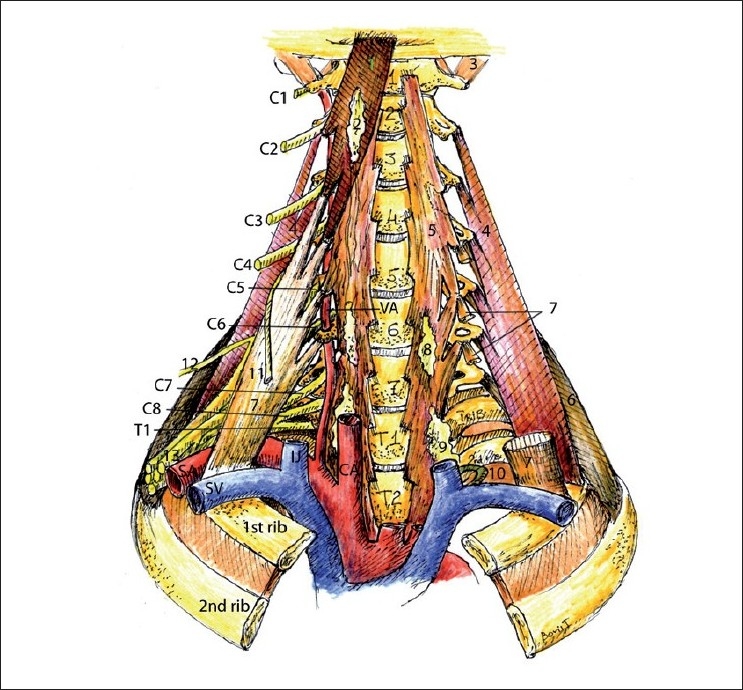
Deep cervical muscles. (1) Longus capitis muscle, (2) superior cervical sympathetic ganglion, (3) rectus capitis muscle, (4) middle scalene, (5) longus colli, (6) posterior scalene muscle, (7) anterior scalene muscle, (8) middle cervical sympathetic ganglion, (9) inferior cervical and upper thoracic sympathetic ganglions, (10) thoracic duct, (11) phrenic nerve, (12) suprascapular nerve, (13) brachial plexus

Longus colli, longus capitis and anterior, middle and posterior scalene muscles [Figure 3].

The longus colli muscle is situated on the anterior surface of the vertebral bodies and is covered by a deep layer of prevertebral fascia. It is approximately 1 cm wide. Injecting LA into the muscle compartment or subfascially at the level of C6 will cause a sympathetic chain and stellate ganglion block because all these structures are inside of this fascial compartment. At a higher level (C2 or C3) the superior cervical sympathetic ganglion or DCP may be blocked by injecting the LA into the longus capitis muscle compartment deep to the deep fascia layer [Figure 3].

There are three scalene muscles: the anterior, middle and posterior scalene muscles. Note the attachments for these muscles [Figure 3] and the relationship of these attachments to the subclavian artery, BP and DCP. The anterior scalene muscle attaches proximally to the anterior tubercles of the transverse processes of C3–C6 and distally to the first rib in front of the subclavian artery, and the middle scalene muscle attaches proximally to the posterior tubercles of the transverse processes of C2–C7 and distally to the first rib posterior to the subclavian artery. The posterior scalene muscle attaches proximally to the posterior tubercles of the transverse processes of C4–C6 and distally to the external border of the second rib [Figure 3].

The spindle shape of the anterior scalene muscle, which is of special note, can be visualized as the US probe slides up the neck (personal observation). It is at its thickest at the level of the cricoid cartilage, but gets thinner as it tracts cephalad and caudad, where it is thin again as it attaches to the first rib. The anterior and middle scalene muscles create the groove or space for the BP and subclavian artery. Injecting LA in this space will provide BP blockade. The anterior scalene does not extend higher than the level of C4 or C3. Above that level, the longus capitis and middle scalene create the groove or paravertebral space for the DCP. It may, therefore, be possible to block the DCP with a single injection at or above the C4 level. Moreover, with a typical C6 level interscalene approach to the BP, substantial cephalad spread is possible along the middle scalene muscle if a large volume of LA is used, which explains the consistent block of the SCP and phrenic nerve with the classic ISB.[18,19]

The phrenic nerve courses downward on the anterior surface of the anterior scalene muscle from the lateral border of the anterior scalene muscle to its medial border behind the subclavian vein into the mediastinum. The phrenic nerve is occasionally visible with US between the sternocleidomastoid and anterior scalene.

As illustrated in Figure 3, the anterior scalene is relatively thin in the middle part of the neck at approximately the level of C5. The phrenic nerve is therefore close to the BP at this point. Anterior spread of LA to the phrenic nerve over the thin anterior scalene muscle at this level could thus be another mechanism of phrenic nerve block during ISB.

To avoid unintentional phrenic nerve blockade during ISB, the BP should be approached as low as possible in the interscalene grove where the phrenic nerve is situated most anterior, further away from the BP. Also, the BP could be approached from the posterior to avoid penetration of the anterior scalene muscle and smaller volumes of LA could be used.

If interscalene BP block is performed lower in the interscalene groove, cephalad spread of LA may also be less prominent, possibly resulting in reduced blockade of the cutaneous nerves of the SCP (supraclavicular nerves responsible for cutaneous innervation of the “shoulder cape”).

The anterior scalene muscle is at its thickest at the level of C6 or C7, and therefore is most suitable for intramuscular injection of paralyzing agents such as botulinum toxin for the possible treatment of neurogenic thoracic outlet syndrome.[20]

The posterior neck muscles, the erectors of the cervical spine, are commonly very tender after any injury or due to tension. When performing posterior approaches to the brachial or cervical plexuses, a muscle-sparing needle trajectory to decrease neck pain should be selected. It is easy to achieve this through the space between the trapezius and the levator scapula muscles for cervical paravertebral block of the brachial or cervical plexuses.[21]

### Superficial muscles

The sternocleidomastoid muscle forms the anterior border of the posterior triangle of the neck. It could be used to rapidly navigate the level of an US scan of the neck; the posterior border of this muscle covers the interscalene groove at Winnie’s point, although at the C3/C4 level its anterior border is close to the transverse process line. The position of the head, especially rotation, affects the relationship of this muscle in regard to deep structures. The omohyoid muscle is another superficial muscle that could be seen with US where it was overlying the BP at the base of the neck.

### Thyroid, Esophagus

These structures are primarily used as important landmarks and need to be recognized to avoid inadvertent damage to them during neural blockades.

**Figure 4:**
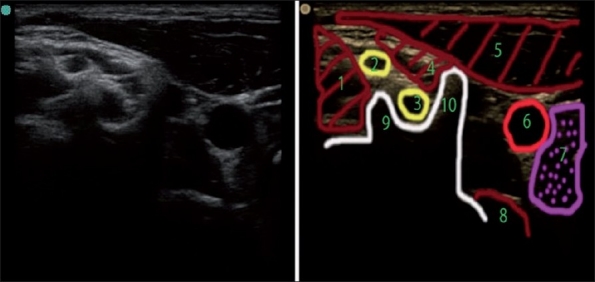
Lateral axial ultrasound neck image at the upper pole of thyroid (C6 vertebral level). (1) Middle scalene, (2) C5 nerve root, (3) C6 nerve root, (4) anterior scalene, (5) sternocleidomastoid, (6) carotid artery, (7) thyroid, (8) longus colli, (9) posterior tubercle of C6, (10) anterior tubercle of C6 (carotid tubercle)

Although not strictly situated in the posterior triangle of the neck, the thyroid gland is easily identifiable and forms a handy landmark for ultrasonography of the neck. The cricoid cartilage is positioned close to the C6 vertebral level and the lower lobe of the thyroid is therefore close to the level of C7–T1 and the upper lobe is close to the level of C5–C6. By sliding the US probe cephalad along the thyroid gland keeping the area that is just lateral to the great vessels in view, the carotid tubercle of C6 can be seen when the thyroid is about to disappear from view [Figure 4].

The recurrent laryngeal nerves are situated between the trachea and the thyroid gland. Because the pretracheal fascia envelops all visceral structures in the anterior neck and provides an extra protection barrier for it, unintentional recurrent nerve blockade occurs most likely at a point where the nerve loops around the subclavian artery on the right side (17 at Figure 4), or it happened due to vagus block more proximally.

The esophagus can be injured during cervical sympathetic block. Asking a patient to swallow helps to pinpoint the esophagus by observing its movement when in doubt. Remember that the esophagus is frequently positioned more to the left of the trachea rather than just behind it.

### Blood vessels

**Figure 5:**
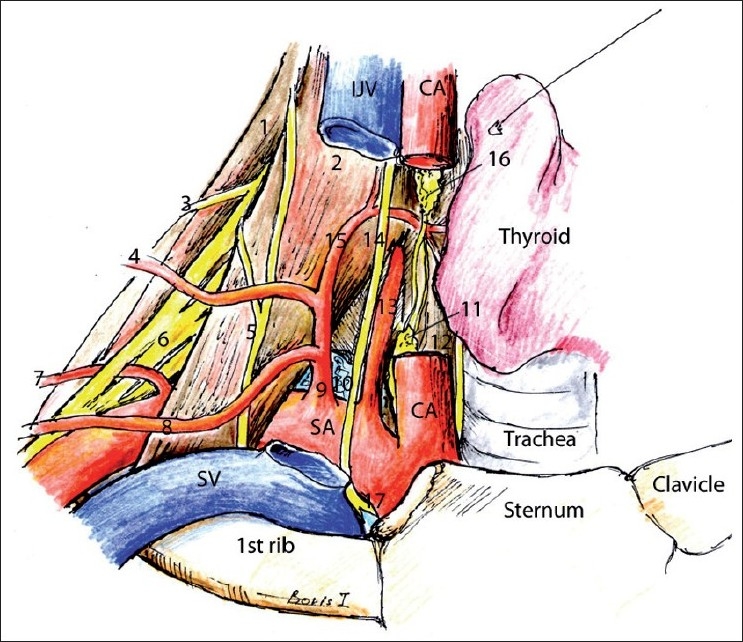
Neck blood vessels. (1) Middle scalene muscle, (2) anterior scalene muscle, (3) dorsal scapular nerve, (4) transverse cervical artery, (5) phrenic nerve, (6) brachial plexus, (7) dorsal scapular artery, (8) suprascapular artery, (9) thyrocervical artery, (10) lung, (11) inferior cervical sympathetic ganglion, (12) longus colli muscle, (13) vertebral artery, (14) vagus, (15) inferior thyroid artery, (16) middle cervical sympathetic ganglion, (17) recurrent laryngeal nerve

Blood vessels [Figure 5] serve as important anatomical landmarks and also should be actively looked for and avoided during needle advancement. The color Doppler function must be used to distinguish nerve roots from arteries because they may look very similar. This is especially true for vertebral artery and thyrocervical trunk at and below the C7 level.

The great vessels: common carotid artery (CCA) and internal jugular vein (IJV), are easy and consistent reference points during neck US scanning. The carotid sheath (14 in Figure 1) envelops the CCA, the IJV and the Vagus nerve.[1] The IJV is collapsible with slight pressure of the US probe or when the patient’s head and torso are elevated. To best visualize the IJV and other veins, the Trendelenberg position could be used or the patient can perform a Valsava maneuver. Valves are frequently visible in the subclavian or innominate veins, which helps to distinguish veins from arteries when transmitted pulsations are present.

The CCA courses from anterior and medial to more posterior and lateral as it ascends cephalad. At the level of C4, the CCA divides into the external and internal carotid arteries, and the bifurcation could be used as a C3–C4-level landmark.[1–4]

The more cephalad portion of the carotid artery is closer to the nerve roots than lower down in the neck. Therefore, if the nerve roots and carotid artery are close to each other on US, it probably indicates a relatively high scanning level.

The vertebral artery (13 in Figure 5) arises from the posterosuperior aspect of the subclavian artery, and in 90% of the people, it enters the transverse foramen of the C6 vertebra as it courses cephalad.[10] The vertebral artery is exposed and could be injured during deep cervical block, cervical sympathetic block and BP block, especially if the last two are performed caudad of the C6 transverse process (at and above the C6 transverse process, the vertebral artery is at least partially protected by bone when it traverses via the transverse foramen). It can be seen below the C6 transverse process slightly anterior to the nerve roots and also between any other transverse processes above C6 in a longitudinal and even in an axial view at any higher level in the neck. As mentioned earlier, the posterior approaches to BP or to DCP are safer in regard to inadvertent injury to the vertebral artery because the vertebral artery is situated medial to the lateral border of the articular column that serves as a protective wall of bones when the needle is advanced from the posterior.

The thyrocervical trunk (9 in Figure 5) is more lateral than the vertebral artery. Both vessels can be seen in an axial view at the base of the neck.

**Figure 6:**
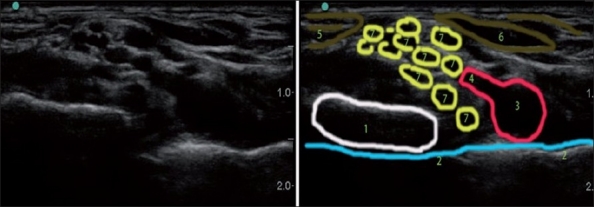
Supraclavicular ultrasound scan demonstrating the dorsal scapular artery. (1) First rib, (2) lung, (3) subclavian artery, (4) dorsal scapular artery, (5) middle scalene muscle, (6) anterior scalene muscle, (7) brachial plexus

The dorsal scapular and, less frequently, the transverse cervical and suprascapular arteries course posterolaterally through the BP. These arteries can be seen during US-assisted supraclavicular BP block [Figure 6].

During cervical sympathetic block, the superior thyroid artery, a branch of the carotid artery, and the inferior thyroid artery, a branch of the thyrocervical trunk, commonly traverse across the needle trajectory and can be seen when color Doppler is used.

Use of color Doppler or power Doppler is advisable to identify small arteries and veins around the intervertebral foramina when a US-guided neuroforaminal epidural or proximal root injection is performed. These are important blood vessels that supply the spinal cord.

On the left side of the lower lateral part of the neck, it is sometimes possible to identify the thoracic lymph duct as it loops from inside out and joins the great veins (10 in Figure 3).

### Lung cupola

US is helpful in visualizing the lung cupola and may aid in avoiding lung and pleural injury. The lower trunk of the BP courses across the lung cupola. The inferior sympathetic ganglion is situated very close to the lung cupola as well. Pneumothorax is a possible complication after any BP blocks above the clavicle and after stellate ganglion block. US use should decrease the risk of pneumothorax and can also be used in the timely diagnosis of pneumothorax.

### Neural structures

Visualization of neural structures in the neck could be used for the diagnosis of several nerve entrapment syndromes, but is more commonly used for US-guided neural blockade.

The echotexture of the proximal nerves is different from the echotexture of the distal nerves.[5,7,8] Roots of the DCP – the anterior rami of the C1–C4 spinal nerves, and the BP – the anterior rami of the C5–T1 spinal nerves, are hypoanechoic and look similar to blood vessels. (Color Doppler can easily distinguish these from blood vessels.) This appearance is not fully understood but could be because of the high ratio of neural tissue (axon-rich fascicles that are anechoic) to nonneural connective tissue (stroma of the nerves). Hyperechoic appearance of the distal peripheral nerves is probably explained by an increased amount of nonneural perifascicular connective tissue that is almost absent in roots that are densely “packed” with axon-rich fascicles.[19] The amount of neural tissue logically decreases as more and more proximal nerve branches take off the spinal nerve as the nerve passes distally. Another explanation could be that spinal nerves as they exit the spine are surrounded by dura and, compared with the peripheral nerves, this dura has not yet migrated into the roots of the BP as it appears more distally to form the septae in the trunks or perineurium of the fascicles in the peripheral nerves.[22,23]

Contrary to the roots or trunks and divisions, the terminal branches of the brachial and cervical plexuses in the neck, such as dorsal scapula nerve, suprascapular nerve, levator scapula nerve, long thoracic nerve, supraclavicular nerves and phrenic nerve, are difficult to identify with US. Their visibility depends on their size, the echocontrast of the surrounding tissues and the quality of the US machine used (and sometimes the experience of the ultrasonographer). The proximity of known structures can provide an “educated guess” to where these nerves should be situated if selective blocks of these nerves are needed.

### Proximal BP sonoanatomy (neck area)

Five roots of the BP form the three trunks.[5,6] Each trunk has two divisions that eventually rearrange into three cords and several distal terminal branches. This most common representation occurs only in about 65% of the people. There are approximately 39 other variations.[5–7,19]

**Figure 7:**
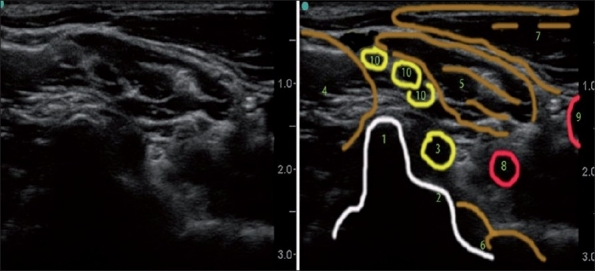
Lateral axial ultrasound neck image at the C7 level. (1) Posterior tubercle of the C7 transverse process, (2) rudimentary anterior tubercle of the C7 transverse process, (3) C7 root, (4) middle scalene muscle, (5) anterior scalene muscle, (6) longus coli muscle, (7) sternocleidomastoid muscle, (8) vertebral artery, (9) carotid artery, (10) brachial plexus (C5–C6 derivates)

Figure 2 depicts the spatial orientation of the BP and offers an explanation of the typical lateral axial US scan through it at the C7 level. See also Figure 7 for the actual US image of this level.

The BP is located between the anterior and the middle scalene muscles and courses posterolaterally to the subclavian artery over the cupola of the lung and the first rib, under the clavicle. Some aberrant BP roots occasionally perforate the anterior scalene muscle proximally and then rejoin the plexus more distally.

Dural sleeves surround the roots of the BP, yet US technology has not developed far enough to allow us to visualize these dural sleeves. In general, therefore, we cannot reliably tell where the roots become trunks and whether the trunks split into the divisions and so on. All proximal blocks should therefore be regarded as root-level blocks or extradural blocks.[22,23]

**Figure 8:**
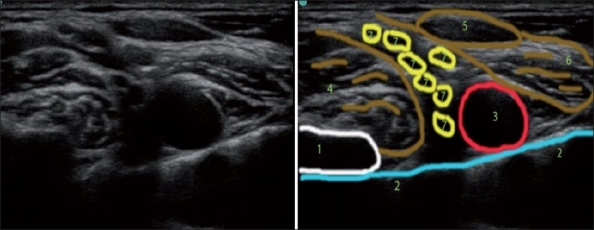
Sagital supraclavicular ultrasound scan. (1) First rib, (2) lung, (3) subclavian artery, (4) middle scalene muscle, (5) omohyoid muscle, (6) anterior scalene muscle, (7) brachial plexus

**Figure 9:**
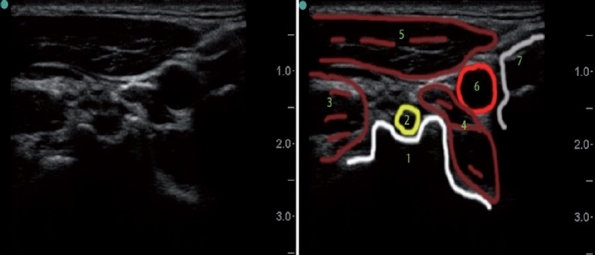
Lateral axial ultrasound neck image at the C4 vertebral level. (1) C5 transverse process, (2) C4 root, (3) middle scalene muscle, (4) anterior scalene/longus coli muscles, (5) sternocleidomastoid muscle, (6) carotid artery, (7) thyroid cartilage

With the US, we can differentiate the root level (C5 versus C6, for example) when tracing a particular root or its derivatives back to the corresponding transverse process (Figure 7 for C7 root and Figure 4 for C6 root), but it is more difficult to do so when we only see five to nine round, hypoanechoic structures one above the other more distal in the interscalene space [Figure 8 for example]. We can usually make an “educated guess” of the identity of the particular “round” neural structure. As depicted in Figure 2, the more caudal in the neck we scan, the more nerves we see; the more proximal roots and their derivatives are situated more superficially and more distal roots are situated deeper on the US picture (one may miss them if US depth is set too shallow). Because of the unpredictable pattern of division of the roots, it is inaccurate to simply count them down assuming that the first most superficial round structure is C5 and the next is C6 and so on. We can confirm our assumption regarding neural structure identity by the appropriate motor response with nerve stimulation or by tracing each structure proximally to the level of the corresponding transverse processes where they initially originate [Figures 4, 7, and 9]. The last maneuver is more reliable.

The ability to differentiate the identity of the neural structures in the neck with US is clinically significant for neural blockade. With any high (C5/C6 cervical level) proximal approach to BP block, there is a challenge to cover a wide anatomical space to achieve a complete block of the entire BP (C5–T1 roots) because those roots are spaced apart. The distance from C5 root to T1 root could be about 7 cm.[6] Figure 2 gives an insight to that challenge if we look at the vertical distance from C5 to C7 and imagine that we trying to cover that area with LA by injecting it from the C5/C6 intervertebral level.

It is well known that with classic ISB that is usually performed at the C6 level, the lower positioned C8 and T1roots will be missed up to 30–50% of the time using the normal volume of LA. If one uses increased volumes of LA or performs the block by using more than one needle position (moving it to the area that is not covered by LA from first injection), a more complete block could be expected. Unfortunately, when we use continuous block and infusion of LA, the limit is 5–10 ml/h and, therefore, it is very important that the tip of the catheter be positioned in the most favorable spot for a particular surgery (C5/C6 root derivatives for shoulder and upper arm surgery; C7 root derivatives for elbow surgery; C8/T1 roots derivatives for wrist and hand surgery). US guidance may be very helpful for this, especially for lower root derivatives that are situated close to the lung cupola and important vascular structures.

As seen in Figure 2, the C5/C6 root derivatives could be blocked at the C6 vertebral level (classic Winnie approach for example) or at the C7vertebrae level or even lower (supraclavicular level for example). The question is if any difference exists based on the point of the blockade. It is logical that if one chooses to block C5/C6 roots and their derivatives very low (below the C7 vertebra), there is a higher chance that some proximal derivatives, such as the suprascapular nerve, could be missed if that particular nerve had already branched off. As was mentioned earlier, blocking the C5/C6 root derivatives at the C6 vertebra level usually provides consistent block of the supraclavicular nerves (C3–C4 derivatives responsible for shoulder cape cutaneous anesthesia) and suprascapular nerve. Unfortunately, phrenic nerve is also inadvertently blocked due to some cephalad and anterior spread of LA.

It seems logical that a block of the same C5/C6 derivatives at the C7 level [Figures 2 and 7] may be the most advantages for optimal analgesic coverage and fewer side-effects. Some studies confirm this notion but more research is needed.[24]

C7 root derivatives could also be blocked at different levels: the C7 transverse process level or lower. Blocking it at the C7 transverse process seems to be quite optimal in regard to the risk of lung and vascular injury (vertebral artery and subclavian artery and its branches). There is no data regarding comparison of that approach with a block that is performed more distally (supraclavicular continuous block for example). Factors unrelated to the quality of the block or risk of immediate complications may determine the choice of one technique over another. Theoretically, geriatric patients with less-spacious and less-compliant plexus sheath in the area between the rib and the clavicle could be more vulnerable to the risk of high BP sheath compartment pressure secondary to continuous infusion via the supraclavicular plexus catheter. Authors of this article believe that this is not the case with more spacious paravertebral space during continuous cervical paravertebral block with the tip of the catheter at the C7 root. This notion needs more research.

### Distal BP sonoanatomy (supraclavicular area)

Tracing the BP with US from the neck more distally, the ultrasonographer gets into the supraclavicular area.

Occasionally, for proximal plexus blocks, it is easier to start US examination from the supraclavicular area where the subclavian artery serves as a consistent reliable US landmark and traces the BP up proximally.

In the supraclavicular area (right above the clavicle), the BP is at its trunk or divisions level (neural structures are still hypoanechoic). The trunks (superior, middle and inferior) are usually vertically positioned one above the other at the lateral side of the interscalene groove and medial border of the first rib. At the lateral border of the first rib, the trunks are usually divided into divisions, but there is a considerable variability of the point of division and BP organization. In general, the BP looks like a “bunch of grapes” located superior and posterolateral to the artery.[25] The sheath-like structure is commonly seen around the BP on US and a distinct “pop” is felt and “seen” on penetration of this structure with the block needle.

In the supraclavicular area, the derivatives of the C5, C6 and C7 roots are situated superior, posterior and lateral to the subclavian artery. Occasionally, but rarely, some of those nerves are located superior and medial to the artery (artery splits the BP into two parts, personal observation). The derivatives of the C8 and T1 roots are consistently located in the corner between the subclavian artery and the lung or the first rib.[25] If the block of those nerves (C8, T1 roots derivatives) is important for a particular procedure, the practitioner should manipulate the US probe (see later) and obtain the image that shows the subclavian artery and the BP “sitting” on the first rib rather than on the lung. When such an image is obtained, the so-called “corner pocket” technique should be used when the needle is advanced in plane with US beam into the corner between the artery and the first rib.[26] In this approach, the first rib serves as a “back stop” to prevent inadvertent pleura or lung injury in case an ultrasonographer fails to accurately visualize the tip of the needle. It is also important to understand that cupola of the lung is medial and one should avoid medial deviation of the needle at all cost. Ideally, the entire needle and the tip should be clearly visualized during the procedure. Injection of the LA in the “pocket corner” reliably blocks derivatives of the lower roots that are commonly missed with more proximal approaches, such as interscalene, and thus this variation of supraclavicular block is optimal for distal arm and hand surgery. With a high volume of LA injected into the “pocket corner,” the entire arm, with the exception of the shoulder, will be blocked. For faster block set up when single injection block is performed, after initial injection of some of the LA in the “corner pocket,” the needle could be repositioned so that the LA is deposited directly around the derivatives of the C5–C7 roots as well.[27]

Occasionally, when the US probe is positioned in the supraclavicular area posterior to the middle third of the clavicle, the subclavian artery is visualized “sitting” on the lung rather than on the first rib. Tilting the US probe as if you are trying to sweep the US plane anteriorly (probe handle moves posterior) is the most common trick that helps to “put the subclavian artery on the rib.” Attention should be paid to correct patient position. When the patient’s shoulder is moved up and backward, the clavicle also moves up and backward, forcing the ultrasonographer to place an US transducer too high and too medial in the supraclavicular area where the subclavian artery is still on the lung cupola medial to the first rib.[28]

The complete BP blockade is common when the block is performed below the C7, right above the proximal part of the first rib slightly more lateral than a typical ISB but more medial than a typical supraclavicular block. Figure 8 demonstrates the US image of this spot. Winnie also described this point in his “subclavian perivascular approach.”[29] At this point, the entire BP is bundled together and could be blocked with a small volume of LA. At this area, attention should be paid to avoid puncturing the lung cupola and the vertebral artery and again US could be very valuable.

### DCP (C1–C4)

The roots of C2, C3 and C4 (the root of C1 is a pure motor nerve) need to be located in order to perform an US-guided block of the DCP. Identification of these roots is based on the identification of the corresponding transverse processes and counting them up from the C7 and C6 transverse processes. Injection of several milliliters of LA at each root or, as mentioned earlier, a single injection at the C3 or C4 level of a larger volume of LA are possible alternatives [Figure 9].[30]

### SCP

We can usually not see the SCP with US but we can see the deep fascia and sternocleidomastoid muscle above it and can “hydrodissect” the appropriate fascial plane with LA at the classic landmark point (see earlier).

The phrenic nerve originates mostly from the C4 [Figure 3], but the sometimes-present accessory phrenic nerve originates lower from the C5 or even the C6.[1] US-guided BP block may decrease the risk of a phrenic nerve block by injecting the LA around the BP, away from the phrenic nerve (avoiding LA “spill” medial and superior to the subclavian artery during supraclavicular block and performing a C5/C6 root derivative block at the C7 transverse process level via a posterior approach).[31] This area needs more research.

The sympathetic ganglions and chain are located deep to the prevertebral layer of the deep fascia on the deep muscles (longus colli and longus capitis) or right on the surface of that fascia (between alar fascia and prevertebral fascia).[1] There are three cervical ganglions: the superior cervical ganglion at the C2 level, the middle cervical ganglion at the C5–C6 level and the lower cervical ganglion at the C7 level [Figure 3]. US visualization of the sympathetic structures is not consistent, but they usually present as hyperechoic spindle-like structures (personal observation). The low cervical ganglion and the upper thoracic ganglion fuse to form the stellate ganglion, which is typically located at the head of the first rib close to the dome of the lung.

Preganglionic sympathetic fibers of the cervical sympathetic chain come from the upper thoracic segments of the spinal cord and the postganglionic grey rami communicantes innervate the upper extremity and head probably by penetrating the deep cervical muscles[32] before joining the BP or entering the cranium along the blood vessels.

The sympathetic chain or ganglions could be blocked intentionally for diagnosis and treatment of sympathetically maintained pain from the arm, head or upper torso or for other indications such as Reynolds syndrome, hyperhydrosis and vascular headaches. To perform this block, LA can be injected under the prevertebral fascia or right above the fascia (between prevertebral and alar fascia)[33,34] or into the belly of the longus colli muscle[30] at the level of C6 (several techniques exist). If LA is injected into the muscle, the longus colli muscle fascial compartment will allow cephalad and caudad spread of LA beyond the injection point. All these approaches obviate the need to perform an injection in the area close to the lung cupola at the level of C7–T1 due to reliable spread of LA along the fascial planes from the C6-level injection. Injecting LA more cephalad (C4 level for example) could be theoretically more selective for upper and middle cervical ganglions, which would be more beneficial for sympathetic block of the cranium rather than the arm.

Occasionally, sympathetic blocks that accompany the BP block are more extensive and cover the unilateral sympathetic innervations of the head, which presents as Horner’s syndrome. It is usually harmless and occurs secondary to extensive spread of a relatively high volume of LA between the deep muscles under the prevertebral fascia in the proximity of the sympathetic chain.

Suprascapular nerve blockade is required for shoulder surgery. It innervates the posterior shoulder capsule, acromio-clavicular joint, coraco-clavicular and coraco-acromial ligaments, the supraspinatus and infraspinatus muscles and the subacromial space. The suprascapular nerve originates from the upper trunk of the BP and is reliably blocked during proximal blocks, such as typical cervical paravertebral block or ISB and, occasionally, during supraclavicular blocks, if LA was deposited more proximally along the upper trunk.

If the suprascapular nerve has not been blocked, or needs to be selectively blocked for diagnostic or therapeutic reasons, it could be blocked more distally, where it passes onto the posterior surface of the scapula and US could be used for this block.[33]

Important cranial nerves such as the vagus, accessory, hypoglossal, glossopharyngeal and facial nerves pass through the neck and can be blocked during different procedures intentionally or unintentionally. As mentioned before, it is difficult to visualize these nerves with US at this level of technological progress.

### Use of the systemic approach versus pattern recognition

**Figure 10:**
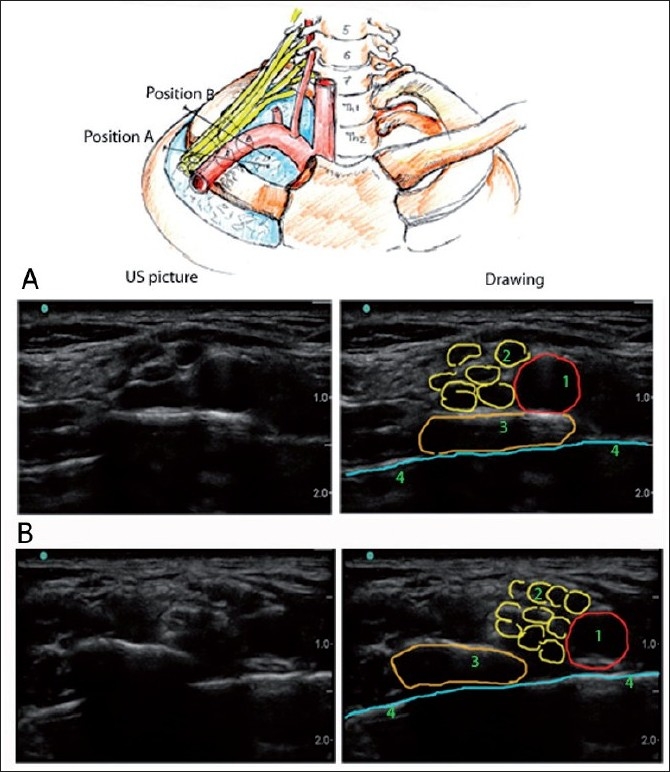
Three-dimensional sonoanatomy of the supraclavicular scans. (1) Subclavian artery, (2) brachial plexus, (3) first rib, (4) pleura/lung

To demonstrate the use of the systemic approach, two supraclavicular ultrasound scans [Figure 10a and b] and underlying 3D anatomy are illustrated. Positioning the probe on line A, just posterior to the clavicle in the coronal plane [Figure 10a] produces the familiar US picture for supraclavicular BP block (pattern recognition for “pocket corner technique”).

It is also possible to place the probe in a slightly different position on line B [Figure 10b]. The probe is now situated more posterior and medial along the axis of the subclavian artery and the first rib. This could be achieved by sliding the probe in this direction or just rotating it clock wise. Tilting the probe handle more anterior may also produce similar results (image B instead of image A). The two images (A and B) may look similar, especially if the depth of the scan is shallow (not like on the illustration where the depth is optimal to see the “step down” from the rib to the pleura).

In position B, however, the artery is situated directly on the pleura of the lung and in position A it is situated on the first rib.

In some patients, due to anatomical variation, a picture similar to Figure 10b would be obtained initially where the subclavian artery is situated on the pleura and not the first rib, even if the probe was placed right behind the clavicle. In this case, tilting the probe handle more posterior may “bring” the artery on the first rib.

Incorrect patient positioning when the shoulders, and therefore the clavicles, are moved too cephalad and backwards might also cause the probe to be “forced” by clavicles into position B rather than position A.

If one fails to recognize that instead of “text book” image A we have image B, and attempts to perform the “corner pocket” block technique in this situation, he or she may cause a pneumothorax.

The ability to interpret the US image correctly, distinguish the hyperechoic line of the rib from the similar-looking line of the pleura, the knowledge of the applied 3D anatomy, in this case the relationships between the clavicle, first rib, the subclavian artery and the lung in the supraclavicular area and the knowledge of how probe manipulation (tilt, rotation and sliding) may help to obtain the correct structures alignment could help to perform the most advantageous US-guided injections and avoid complications.
